# Thermally Stable Wireless Patch Antenna Sensor for Strain and Crack Sensing

**DOI:** 10.3390/s20143835

**Published:** 2020-07-09

**Authors:** Dan Li, Yang Wang

**Affiliations:** 1 School of Civil and Environmental Engineering, Georgia Institute of Technology, Atlanta, GA 30301, USA; dli323@gatech.edu; 2 School of Electrical and Computing Engineering, Georgia Institute of Technology, Atlanta, GA 30301, USA

**Keywords:** patch antenna sensor, RFID, thermal stability, energy harvesting, strain, crack, wireless sensing

## Abstract

Strain and crack are critical indicators of structural safety. As a novel sensing device, a patch antenna sensor can be utilized to wirelessly estimate structural strain and surface crack growth through resonance frequency shift. The main challenges for the sensor are other effects such as temperature fluctuation that can generate unwanted resonance frequency shift and result in large noise in the measurement. Another challenge for existing designs of patch antenna sensor is the limited interrogation distance. In this research, thermally stable patch antenna sensors are investigated for more reliable measurement. Fabricated on a substrate material with a steady dielectric constant, a new passive (battery-free) patch antenna sensor is designed to improve reliability under temperature fluctuations. In addition, another newly designed dual-mode patch antenna sensor is proposed to achieve a longer interrogation distance. Extensive experiments are conducted to characterize the patch antenna sensor performance, including thermal stability, tensile strain sensing, and emulated crack sensing. The two new patch antenna sensors are demonstrated to be effective in wireless strain and crack measurements and have potential applications in structural health monitoring (SHM).

## 1. Introduction

Civil structures, such as buildings and bridges, can suffer from damage caused by various types of loads during the life span. Without proper maintenance, the damage can adversely affect the performance of a structure and may even result in structural failure [1]. Structural health monitoring (SHM) systems can advance time-based maintenance into more effective condition-based maintenance by periodically measuring structural responses [2,3]. Among the measurements, strain is often an important indicator of stress concentration and crack development. Traditional strain measurements usually rely on metal foil strain gages and fiber optical sensors [4]. However, these sensing technologies require lengthy cables for data acquisition and power supply, which increase the overall installation and maintenance cost of the whole monitoring system [5,6].

The development of wireless communication technology has facilitated a more convenient application of SHM systems on large structures. A typical wireless sensing system consists of a server and multiple sensor nodes. The server coordinates the wireless sensing network and receives measurement data from all the sensor nodes. Each sensor node usually has at least an embedded processor and a wireless transceiver. The advantageous features of onboard computing and wireless communication enable the dense and rapid deployment of sensor nodes. During past decades, various wireless sensing systems have been developed for SHM. For example, a low-cost wireless modular monitoring system was first proposed by Straser and Kiremidjian [7]. Among others, Lynch et al. [8] and Wang et al. [9] developed wireless sensing platforms that are validated on numerous civil structures. Kane et al. [10] later proposed an extensible dual-core wireless sensing node, named Martlet, which provides more powerful onboard processing [11]. The Mote series (Imote and Imote2) wireless sensing systems have also been deployed for full-scale monitoring of civil infrastructures [12,13,14]. Extensive literature reviews about wireless sensing systems related to SHM applications can be found in [15,16]. Nevertheless, although the wireless sensing systems have achieved success in field deployment, the requirement of onboard battery power remains a difficulty for long-term applications [17]. Many sensor locations on a large structure may not have a reliable source for energy harvesting. Even with reliable solar power, rechargeable batteries may need frequent replacement when operating in the outdoor environment.

To address the challenge of power source, passive (battery-free) wireless sensors have been proposed and studied. With the integration of technologies such as near field communication (NFC) and radiofrequency identification (RFID), passive wireless sensors harvest energy from the interrogation signal and do not require an onboard power supply. Among various passive wireless sensors for strain measurement, antenna sensors stand out for its simple configuration and low cost [17]. The sensing mechanism relies on the fact that the electromagnetic resonance frequency of an antenna depends on its dimension [18]. Based on this physics principle, the wirelessly identified resonance frequency shift of an antenna sensor can be utilized to estimate the strain applied to it. Due to the lack of onboard power, various signal modulation methods for passive antenna sensors have been investigated to increase the reliability of wireless communication. Deshmukh and Huang [19] proposed to use light-activated microwave impedance switch to change the phase of the antenna backscattering signal, which can be distinguished from environmental reflections. The frequency doubling technique using a Schottky diode is another signal modulation method for passive antenna sensors [20]. In addition, RFID technology provides a convenient way to modulate the response signal of passive antenna sensors [21]. Finally, more literature reviews on the passive antenna sensor for strain measurement can be found in survey articles [3,22].

Although extensive simulations and experiments have validated the strain and crack sensing performance of passive antenna sensors, thermal stability and limited interrogation range remain as major challenges for reliable measurement. Besides structural strain, other environmental disturbances, such as temperature fluctuation, can also result in the resonance frequency shift of the antenna by changing the dielectric constant of antenna substrate. A large frequency shift due to temperature fluctuation brings difficulty in distinguishing strain and crack effects from the temperature effect. To improve the strain and crack sensing performance during temperature fluctuation, this research proposes RFID antenna sensors with a thermally stable substrate material. Another limitation of the passive antenna sensor is the relatively short interrogation distance, due to lack of onboard power source. Some researchers have investigated techniques to increase the interrogation distance of RFID-based strain sensors [23,24]. Nevertheless, the performance of wireless measurement over long distances needs to be further validated through experiments. In this paper, we study the design of a dual-mode patch antenna sensor, which can automatically switch between passive and active modes. Toward the dual-mode operation, the patch antenna sensor is attached to a power management circuitry. Incorporated with a solar cell, the circuitry can charge a coin cell battery and actively power the RFID chip when sunlight is available. In this active mode, the sensor requires less radiofrequency (RF) power for responding to reader interrogation, and thus achieves longer interrogation distance. On the other hand, when the battery power is depleted, or the battery dies from a long-time outdoor operation, the sensor can still operate in passive mode, solely relying on the RF power from the reader interrogation. In summary, the dual-mode patch antenna sensor maintains the reliability of passive wireless sensors while providing the advantage of the long interrogation distance of active wireless sensors.

The rest of the paper is organized as follows. Section 2 describes the designs of two thermally stable patch antenna sensors, including the passive patch antenna sensor and the dual-mode patch antenna sensor. Section 3 introduces the strain and crack sensing mechanism of the patch antenna sensors, and the wireless sensing system for the RFID based antenna sensors. Section 4 presents the outdoor temperature experiment for validating the thermal stability of the passive antenna sensor. Section 5 describes the tensile test results for evaluating the strain sensing performance of the dual-mode sensor in both the passive and active modes. Section 6 shows the experimental results illustrating the crack sensing performance of the passive antenna sensor. Section 7 provides a summary and future work.

## 2. Patch Antenna Sensor Designs

Among several types of antennas that can be used as strain and crack sensor, patch antenna and its variations are simple and low-profile. Previous research work has proposed the patch antenna sensor with RT/duroid^®^ 5880 (manufactured by Rogers Corporation, Chandler, AZ, USA) substrate [21]. Although the sensor has shown good performance for wireless strain and crack sensing, research has proven that under temperature fluctuation, the design with RT/duroid^®^ 5880 substrate undergoes large resonance frequency change, due to its large dielectric constant variation (approximately 125 ppm/°C) [25]. To improve sensor reliability to temperature fluctuation, a thermally stable substrate material RT/duroid^®^ 6202 (manufactured by Rogers Corporation, Chandler, AZ, USA) is chosen as the new antenna substrate material. As the substrate provides more stable dielectric constant under temperature fluctuation, the patch antenna sensor is expected to have more consistent resonance frequency when temperature fluctuates. In addition, integrated with energy harvesting technology, a dual-mode patch antenna sensor is designed to achieve longer interrogation distance. Section 2.1 presents the design of the passive patch antenna sensor for strain and crack sensing. Section 2.2 shows the design of the dual-mode patch antenna sensor.

### 2.1. Passive Patch Antenna Sensor

This section describes the passive patch antenna sensor design. The sensor adopts RT/duroid^®^ 6202 as substrate material which has a more stable dielectric constant under temperature fluctuation. The material RT/duroid^®^ 6202 is a poly-tetra-fluoro-ethylene (PTFE) composite with limited woven glass reinforcement. The low dielectric constant ($\beta_{r}=$ 2.90) offers suitable electrical property for antenna sensor design. The low thermal coefficient of dielectric constant (5 ppm/°C) improves the sensor reliability when the ambient temperature fluctuates. The thickness of the substrate is 30 milli-inches which balances the interrogation range and strain transfer ratio, i.e., the ratio of the strain on the top copper cladding of the antenna over the strain on the base structure.

The SL3S1013 RFID chip (manufactured by NXP Semiconductors, Eindhoven, Netherlands) is chosen for wireless communication. The RFID chip contains a 96-bit tag identifier including a 48-bit factory locked unique serial number. Equipped with an advanced anti-collision mechanism, the RFID chip enables the reader to simultaneously access multiple antenna sensors nearby. The small footprint (0.0394 × 0.0571 inches^2^) of the RFID chip further reduces the total size of the antenna sensor. The broad operation frequency range (from 840 MHz to 960 MHz) of the RFID chip facilitates international usage. The chip’s high sensitivity and low power design provide a long interrogation range of the passive patch antenna sensor. In addition, the compatibility with external power enables the design of a dual-mode patch antenna sensor, which achieves a longer interrogation range and is described in Section 2.2.

**Figure 1:**
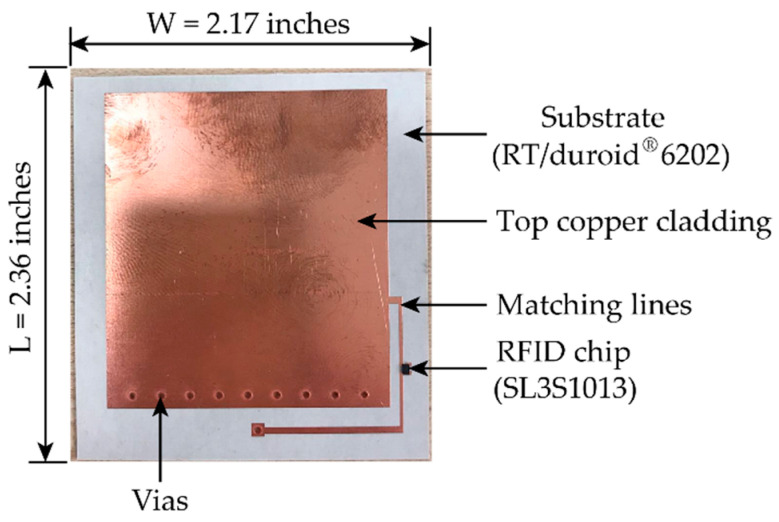
A prototype of the passive patch antenna sensor.

Figure 1 shows the front view of the passive patch antenna sensor. The total size of the antenna sensor is 2.17 × 2.36 inches^2^. The sensor adopts a quarter-wave rectangular patch (folded-patch) antenna topology [26]. The top copper and the ground plane are connected through vias, allowing about a 50% reduction to the footprint size of the patch antenna. The length of the top copper cladding is designed so that the resonance frequency of the antenna sensor achieves about 900 MHz RFID band. To enable efficient power transfer, the lengths of matching lines are tuned to achieve impedance matching between the RFID chip (21.2 − *j*199.7 Ω in passive mode [27]) and the patch antenna.

### 2.2. Dual-Mode Patch Antenna Sensor

**Figure 2:**
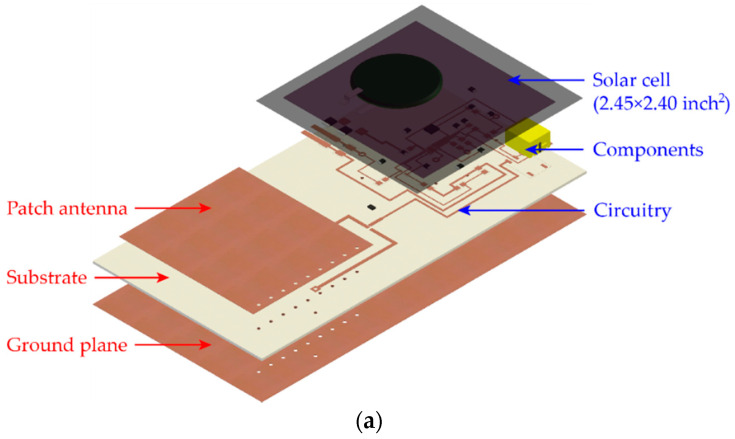

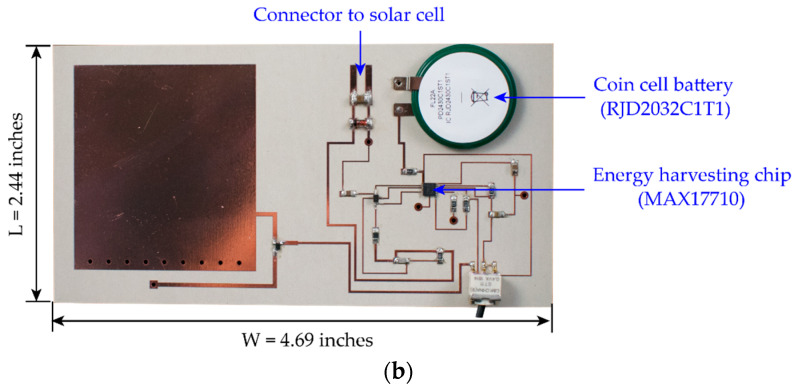
Dual-mode patch antenna sensor: (**a**) design drawing, (**b**) picture of dual-mode patch antenna sensor (solar cell removed).

This section describes the dual-mode patch antenna sensor design. Despite the advantages of operating without an onboard power supply, the passive patch antenna sensor can only support limited interrogation range. To increase the interrogation distance, a dual-mode RFID antenna sensor is designed. Figure 2 shows the design drawing and picture of the prototype dual-mode patch antenna sensor. The total size of the antenna sensor is 2.44 × 4.69 inches^2^. Both the drawing and picture show that a patch antenna sensor and a power management circuitry are fabricated on the same piece of substrate. Equipped with the power management circuitry, this sensor can harvest solar energy, storing energy in a rechargeable coin battery, and operating in active mode. When the battery runs out, this dual-mode RFID patch antenna sensor can automatically fall back to passive mode.

The patch antenna sensor is designed similarly as described in Section 2.1. The matching lines are redesigned for efficient power transfer between the RFID chip and the patch antenna. In active mode, the RFID chip operates with an impedance of 6.9 − *j*205.5 Ω [27]. The designed matching lines must balance the impedance matching between passive mode and active mode and ensure the patch antenna to operate reliably at about 900 MHz for both modes.

The power management circuitry derives energy from solar radiation and supplies regulated voltage to the RFID chip. The energy harvesting IC (integrated circuit) chip MAX17710 (manufactured by Maxim Integrated, San Jose, CA, USA) is adopted for this application. This IC chip both charges a cell battery with over-charge protection and powers the RFID chip with over-discharge protection. The harvested solar energy is stored in a high-capacity Lithium-Ion rechargeable coin cell battery RJD2032C1T1 (manufactured by Illinois Capacitor, Des Plaines, IL, USA), which improves the reliability of the patch antenna sensor.

## 3. Wireless Sensing and Measurement Mechanism

The strain/crack induced resonance frequency shift of the antenna sensor can be wireless detected and utilized to estimate the structural strain and surface crack propagation. Section 3.1 introduces the sensing mechanism of the patch antenna sensor. Section 3.2 describes the wireless measurement mechanism of the RFID based patch antenna sensing system.

### 3.1. Sensing Mechanism

**Figure 3:**
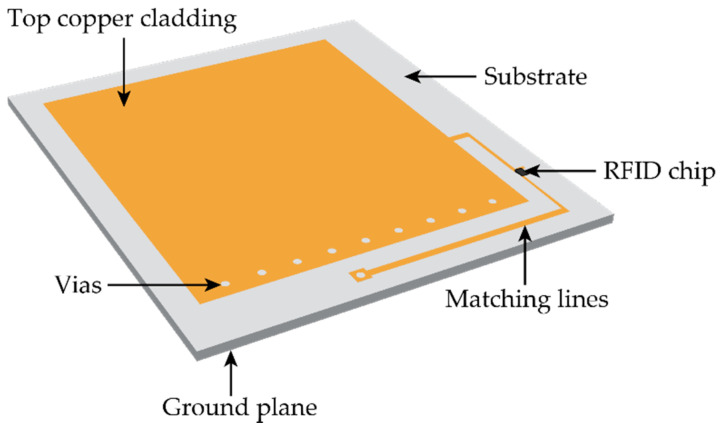
Illustration of the patch antenna sensor.

This section describes the sensing mechanism of patch antenna sensors. A patch antenna sensor can wirelessly measure strain and/or crack on a structural surface through the shift of its electromagnetic resonance frequency. Figure 3 illustrates the patch antenna sensor design. The RFID chip is mounted on the top side of the dielectric substrate and used for wireless communication. The matching lines are designed to achieve the best impedance matching between the RFID chip and the antenna.

According to electromagnetic theory, the resonance frequency $f_{R}$ of a folded patch antenna at certain temperature T can be calculated as:

(1) $$f_{R}\left(0,T\right)=\frac{c}{4L\sqrt{\beta_{r}\left(T\right)}}$$

where c is the speed of light; L is the length of the patch antenna; $\beta_{r}$ is the effective dielectric constant of the substrate depending on temperature T.

When strain ε is applied, the length of the patch antenna changes from L to L(1+ε). In SHM application on civil structures, the dimensionless ε is usually on the order of 10^−5^ (10 micro-strains) to 10^−3^ (1000 micro-strains). If the effective dielectric constant of the substrate remains as constant, the change of resonance frequency Δf of the patch antenna due to strain ε can be calculated as

(2) $$\Delta f\left(T\right)=f_{R}\left(\varepsilon ,T\right)-f_{R}\left(0,T\right)=\frac{f_{R}\left(0,T\right)}{1+\varepsilon }-f_{R}\left(0,T\right)\approx -f_{R}\left(0,T\right)\varepsilon =S\left(T\right)\varepsilon$$

Here S(T) is the theoretical strain sensitivity of the folded patch antenna sensor at temperature T. As shown in Equation (2), at a constant ambient temperature, the change of resonance frequency Δf of the patch antenna has an approximately linear relationship with strain ε, especially when the strain ε is small. This approximately linear relationship indicates that by wirelessly measuring the antenna resonance frequency, the applied strain can be derived.

### 3.2. Wireless Measurement Mechanism

**Figure 4:**
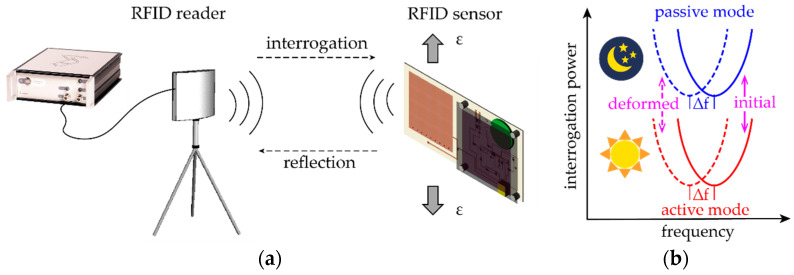
Illustration of the wireless sensing system for the patch antenna sensor: (**a**) wireless sensing system, (**b**) interrogation power curve.

This section describes the wireless measurement mechanism of the RFID based patch antenna sensor. To wirelessly obtain the resonance frequency, an RFID reader Tagformance Lite (manufactured by Voyantic Ltd., Espoo, Finland) is used to interrogate the patch antenna sensor. Figure 4 shows an overview of the wireless sensing system composed of an RFID reader and a dual-mode patch antenna sensor. Upon measurement, the reader emits an RF interrogation signal to the sensor at a specific frequency. The patch antenna sensor captures the signal and transmits the energy to the RFID chip. When the captured power reaches the activation threshold, the RFID chip modulates and sends the reflection signal to the reader. After receiving the reflection signal, the reader records the transmitted power and repeats the same procedure at the next frequency point. The interrogation power curve (Figure 4b) can be obtained once the reader sweeps through the interested frequency band. When the sensor operates in passive mode, the RF signal from the reader is the only power source for exciting the RFID chip, and thus the sensor needs relatively high interrogation power for wireless communication (blue curves in Figure 4b). When in active mode with onboard battery power, excitation of the RFID chip requires less interrogation power (red curves in Figure 4b). The resonance frequency of the patch antenna sensor refers to the frequency at which the minimum amount of interrogation power is required to excite the RFID chip. When the antenna sensor deforms together with based structure, the resonance frequency shifts according to the structural strain.

## 4. Thermal Stability Test

Thermal stability of the patch antenna sensor is investigated through an outdoor temperature test (Section 4.1) and a temperature chamber test (Section 4.2).

### 4.1. Outdoor Test

**Figure 5:**
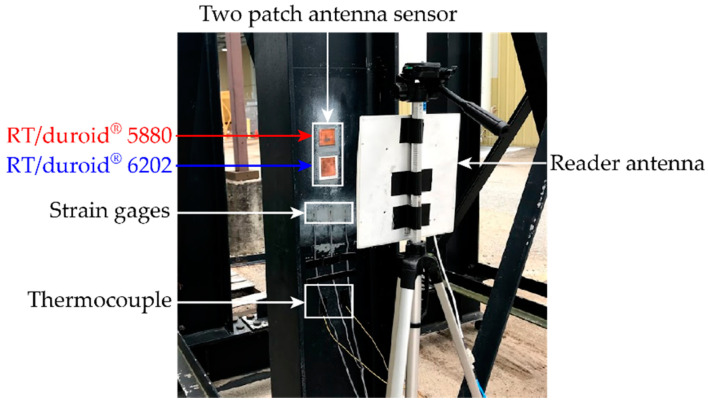
Experimental setup for outdoor temperature test.

An outdoor temperature test is conducted to study the influence of the temperature fluctuation on the resonance frequency of the patch antenna sensors. Figure 5 shows the outdoor experiment setup. Two antenna sensors, one with RT/duroid^®^ 5880 substrate and the other with RT/duroid^®^ 6202 substrate, are installed on the web surface of a steel I-section column. Metal foil strain gages are installed to measure the temperature-induced strain on the steel column surface. To keep track of temperature fluctuations in the field, two thermocouples are installed near the sensors. A reader antenna is placed 12 inches away from patch antenna sensors for wireless interrogation.

**Figure 6:**
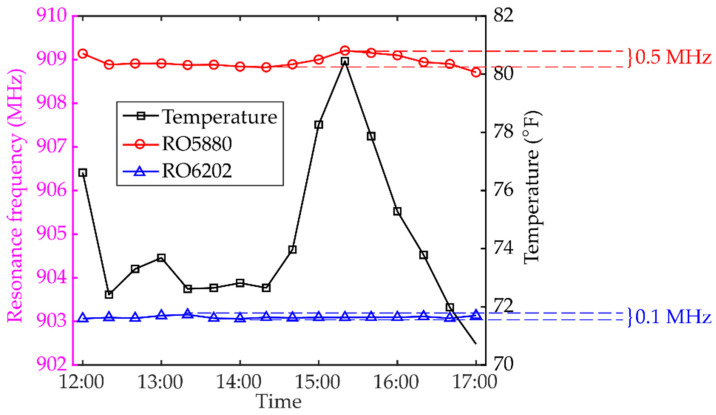
Resonance frequency change due to temperature fluctuation.

The outdoor test starts at noon with ambient temperature at around 76 °F. The temperature is measured every 20 min. until 17:00. The temperature fluctuation is plotted in Figure 6. The highest temperature is around 80 °F and the lowest temperature is around 70 °F. At each time step, the interrogation power threshold is measured for both patch antenna sensors. To reduce measurement noise, the reader antenna sweeps through the target frequency span five times for each measurement. Then the average among the five interrogation power threshold curves is calculated. Resonance frequencies of both patch antenna sensors are extracted from the average interrogation power threshold curves and shown in Figure 6. A total of ~0.5 MHz resonance frequency change is observed on the patch antenna sensor with RT/duroid^®^ 5880 substrate during the test. Meanwhile, the patch antenna senor with RT/duroid^®^ 6202 substrate shows a total of ~0.1 MHz resonance frequency change, which is much less than that of the previous design with RT/duroid^®^ 5880 substrate.

### 4.2. Chamber Test

**Figure 7:**
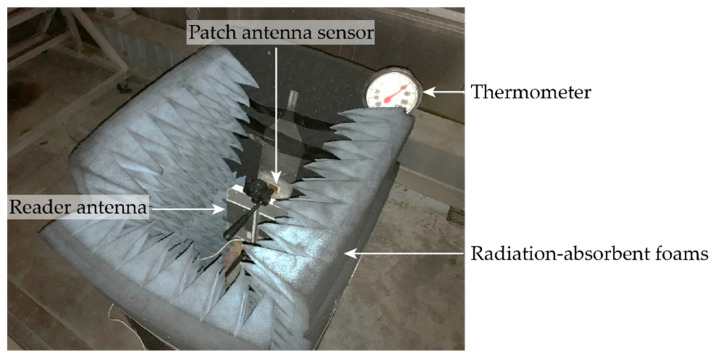
Experimental setup for the temperature chamber test.

A temperature chamber test is conducted to further investigate the thermal effects on the resonance frequency change of the patch antenna sensor with substrate material RT/duroid^®^ 6202. Figure 7 shows the experimental setup for the temperature chamber test. Installed on an aluminum plate, a passive patch antenna sensor is placed in the temperature chamber. A reader antenna is placed facing the sensor for wireless interrogation. Surrounding the sensor and the reader are radiation-absorbent foams which serve to reduce the electromagnetic interference caused by the electromagnetic wave reflection from metal surfaces in the chamber. A thermometer is placed nearby the sensor, as shown in Figure 7, to measure the temperature changes.

**Figure 8:**
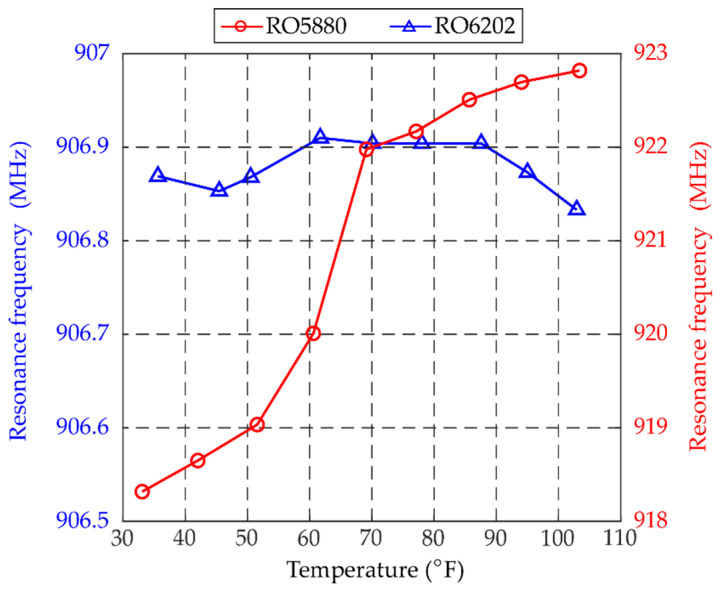
Resonance frequency change during temperature chamber test.

The chamber is heated to 101 °F at the beginning of the test and gradually cooled down to 37 °F. A total of 9 temperature levels are tested. After the temperature becomes stable at each level, five frequency sweeps are conducted to measure the interrogation power threshold of the patch antenna sensor. The resonance frequency of the antenna sensor at each temperature level is extracted from the averaged interrogation power threshold curve. Figure 8 shows the resonance frequency change of the patch antenna sensor with substrate material RT/duroid^®^ 6202. For comparison, the resonance frequencies of a patch antenna sensor with substrate material RT/duroid^®^ 5880 during temperature fluctuation are plotted in the same figure [25]. A total of ~0.1 MHz resonance frequency change is observed on the patch antenna senor with RT/duroid^®^ 6202 substrate when the chamber temperature reduces from 101 °F down to 37 °F. On the other hand, the patch antenna senor with RT/duroid^®^ 5880 substrate experiences ~5 MHz resonance frequency change when the temperature changed from 103 °F to 33 °F. The comparison between two different types of substrate materials indicates that the antenna sensor with RT/duroid^®^ 6202 substrate provides much better thermal stability.

## 5. Strain Sensing Test

**Figure 9:**
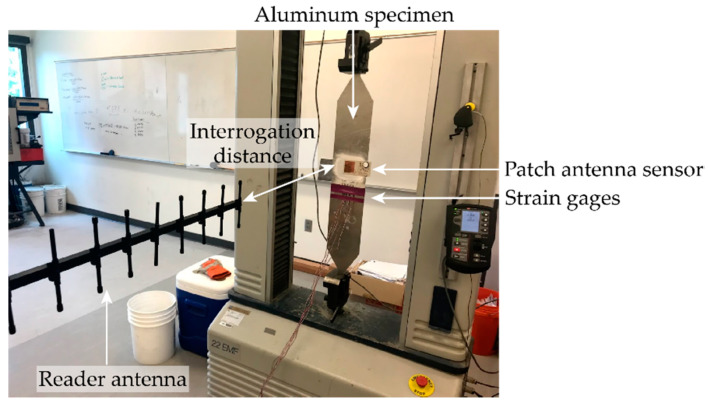
Experimental setup for the tensile test.

Laboratory tensile tests are conducted to evaluate the strain sensing performance of the dual-mode patch antenna sensor. Figure 9 shows the experimental setup for the tensile test on the dual-mode patch antenna sensor. The patch antenna sensor and reference metal foil strain gages are installed in the middle of an aluminum specimen. A Yagi antenna is used as the interrogation reader antenna. The interrogation distance between the patch antenna sensor and the reader antenna is set as 36 inches for passive mode test and 60 inches for active mode test.

### 5.1. Passive Mode Test

**Figure 10:**
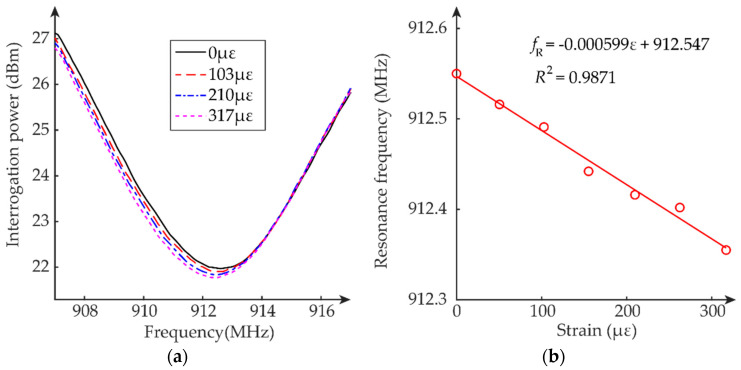
Tensile test results of passive mode test: (**a**) average interrogation power threshold, (**b**) resonance frequency $f_{R}$ versus strain ε.

This section describes the tensile strain sensing performance of the dual-mode patch antenna sensor working in passive mode. The load applied by the tensile machine is configured so that approximately each loading step generates a 50 με strain increment. From 0 to about 300 με, a total of seven loading steps are tested. The interrogation power threshold of the patch antenna sensor is measured at each loading step. For each measurement, again five frequency sweeps are conducted for averaging. The average interrogation power threshold curves at different loading steps/strain levels are plotted in Figure 10a. For clarity, the figure only plots the interrogation power curves of four strain levels. The test results show that the interrogation power for passive mode is from 22 dBm to 27 dBm.

The resonance frequency of the patch antenna sensor at each strain level is determined by peak picking of each average interrogation power threshold curve. As expected, the resonance frequency decreases as the tensile strain increases. Figure 10b plots the resonance frequency change with the strain. Linear regression is applied to these data points, and the strain sensitivity is calculated as −599 Hz/με. In addition, the coefficient of determination is 0.9871, confirming the approximately linear relationship between resonance frequency and strain.

### 5.2. Active Mode Test

**Figure 11:**
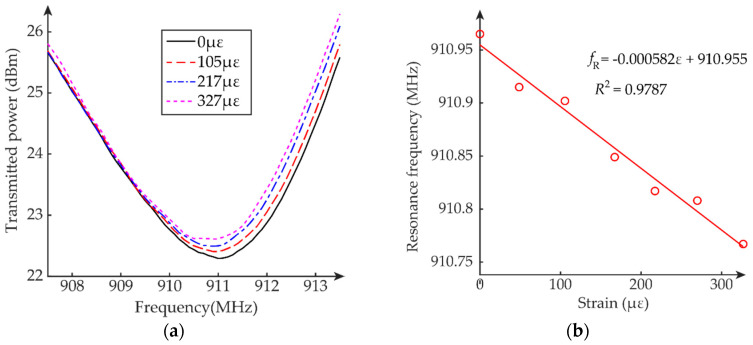
Tensile test results of active mode test: (**a**) average interrogation power threshold, (**b**) resonance frequency $f_{R}$ versus strain ε.

This section describes the tensile strain sensing performance of the dual-mode patch antenna sensor working in active mode. The interrogation distance is increased to 60 inches, about 1.67 times longer than that of the passive mode test. All the other experimental setup and data analysis methods are the same as before. The average interrogation power threshold curves at different loading steps/strain levels are plotted in Figure 11a. For clarity, the figure only plots the interrogation power curves of four strain levels. The test results show that the interrogation power for active mode is from 22 dBm to 26 dBm, which is like the power level for passive mode as shown in Section 5.1. This confirms that using a similar amount of interrogation power, the patch antenna sensor achieves longer interrogation distance at active mode.

The resonance frequency of the patch antenna sensor at each strain level is determined by peak picking of each average interrogation power threshold curve. As expected, the resonance frequency decreases as the tensile strain increases. Figure 11b plots the resonance frequency change with the strain. Linear regression is applied to these data points, and the strain sensitivity is calculated as −582 Hz/με. In addition, the coefficient of determination is 0.9787, confirming the approximately linear relationship between resonance frequency and strain.

## 6. Crack Sensing Test

**Figure 12:**
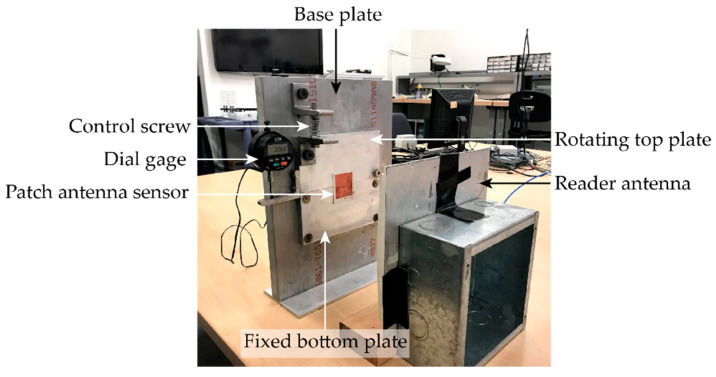
Experimental setup for the emulated crack test.

To investigate the crack sensing performance of the patch antenna sensor, an emulated crack test is carried out. Figure 12 shows the crack test setup, including a crack testing device which is designed for emulating crack propagation. The crack testing device consists of three aluminum plates, i.e., a base plate, a rotating top plate, and a fixed bottom plate. The fixed bottom plate is fastened to the base plate by four corner bolts. The rotating top plate is attached to the base plate by one bolt at the bottom right corner, which acts as the rotation axis. All three plates are thick enough and can be assumed to remain rigid during crack testing for the sensor. A pair of angle brackets are fastened on the rotating top plate and the base plate. By turning a fine-resolution control screw between the angle brackets, the top plate rotates along with the bolt and a crack/gap is opened between the top and bottom plates. With the probe pushing against an angle bracket attached to the top plate, a digital dial gauge (0.0001 in. resolution) is used to measure the crack opening size.

For crack sensing, the antenna sensor is bonded on the rotating and fixed plates, above the gap and at the center of the crack opening line, as shown in Figure 12. The panel reader antenna faces the center of the patch antenna sensor at 12 inches. At each gap opening size, the Tagformance reader sweeps through a frequency band and measures the interrogation power threshold, so that the resonance frequency of the antenna sensor can be determined. After the reader finishes interrogation at one gap opening size, the displacement control screw is turned to reach the next gap opening size and the measurement is repeated.

**Figure 13:**
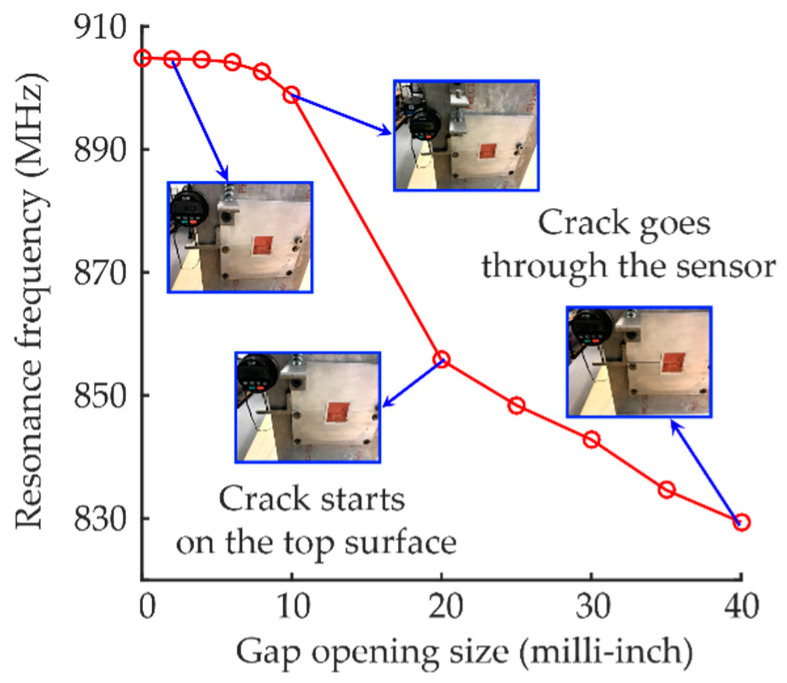
Resonance frequency $f_{R}$ versus gap opening size at dial gage.

In total, eleven gap opening sizes are wirelessly measured during the experiment. The resonance frequencies extracted from interrogation power curves are plotted in Figure 13. For clarity, only four representative photos of the deformed/cracked antenna sensor are shown in the plot. No fracture but slight deformation occurs on the sensor when the gap opening size is smaller than 10 milli-inches. When the gap opening size is larger than 20 milli-inches, the crack starts to propagate on the top surface of the sensor. After the gap opening size reaches 40 milli-inches, the crack grows through the entire antenna width and no response from the antenna sensor can be received by the Tagformance reader.

## 7. Summary and Future Work

In this paper, thermally stable patch antenna sensors have been designed and validated for monitoring strain and crack growth of civil structures. Extensive experiments lead to the following conclusions. The temperature-induced dielectric constant change can result in unwanted resonance frequency change in patch antenna sensors. The thermally stable substrate material can improve the strain and crack sensing performance of patch antenna sensors by preserving the dielectric constant at different temperature levels. The tensile test results demonstrate that the developed patch antenna sensors can measure small structural strain by wireless identifying the resonance frequency shift. In addition, working in active mode, the dual-mode patch antenna sensor can achieve longer interrogation distance with the assistance of solar-charged battery power. The emulated crack sensing test results show that the designed patch antenna sensor is capable of tracking surface crack propagation. As the crack grows, the resonance frequency of the patch antenna sensor decreases as expected.

Future research can investigate calibration according to temperature effects to further eliminate the unwanted uncertainties in the strain and crack measurements. Sensor performance may also be fine-tuned through more detailed multiphysics modeling and simulation.
